# Metabolic instruction of the graft-versus-leukemia immunity

**DOI:** 10.3389/fimmu.2024.1347492

**Published:** 2024-03-04

**Authors:** Ann-Cathrin Burk, Petya Apostolova

**Affiliations:** ^1^ German Cancer Consortium (DKTK), partner site Freiburg, a partnership between DKFZ and Medical Center - University of Freiburg, Freiburg, Germany; ^2^ Department of Medicine I, Medical Center – University of Freiburg, Faculty of Medicine, University of Freiburg, Freiburg, Germany; ^3^ Department of Biomedicine, University Hospital Basel and University of Basel, Basel, Switzerland; ^4^ Division of Hematology, University Hospital Basel, Basel, Switzerland

**Keywords:** allogeneic hematopoietic cell transplantation, graft-versus-leukemia effect, metabolism, acute myeloid leukemia, T cells, anti-tumor immunity

## Abstract

Allogeneic hematopoietic cell transplantation (allo-HCT) is frequently performed to cure hematological malignancies, such as acute myeloid leukemia (AML), through the graft-versus-leukemia (GVL) effect. In this immunological process, donor immune cells eliminate residual cancer cells in the patient and exert tumor control through immunosurveillance. However, GVL failure and subsequent leukemia relapse are frequent and associated with a dismal prognosis. A better understanding of the mechanisms underlying AML immune evasion is essential for developing novel therapeutic strategies to boost the GVL effect. Cellular metabolism has emerged as an essential regulator of survival and cell fate for both cancer and immune cells. Leukemia and T cells utilize specific metabolic programs, including the orchestrated use of glucose, amino acids, and fatty acids, to support their growth and function. Besides regulating cell-intrinsic processes, metabolism shapes the extracellular environment and plays an important role in cell-cell communication. This review focuses on recent advances in the understanding of how metabolism might affect the anti-leukemia immune response. First, we provide a general overview of the mechanisms of immune escape after allo-HCT and an introduction to leukemia and T cell metabolism. Further, we discuss how leukemia and myeloid cell metabolism contribute to an altered microenvironment that impairs T cell function. Next, we review the literature linking metabolic processes in AML cells with their inhibitory checkpoint ligand expression. Finally, we focus on recent findings concerning the role of systemic metabolism in sustained GVL efficacy. While the majority of evidence in the field still stems from basic and preclinical studies, we discuss translational findings and propose further avenues for bridging the gap between bench and bedside.

## 1 Introduction

Allogeneic hematopoietic cell transplantation (allo-HCT) is historically one of the earliest routinely used forms of cancer immunotherapy (1, 2). Its application has been increasing worldwide since the 1980s (3). To date, allo-HCT is the only curative treatment option for patients with highly aggressive hematological malignancies, such as adverse-risk acute myeloid leukemia (AML). However, more than 30% of the patients with AML would eventually relapse after allo-HCT (4). Patients with relapse can be treated either with chemotherapy or with immune cell-directed therapies aiming at reinvigorating the GVL response (5). One such immunotherapy is the administration of donor lymphocyte infusions (DLI), but the response rates in AML are modest at approximately 20% (6, 7). Other strategies to boost the anti-leukemic immune response include immune checkpoint inhibitors and flotetuzumab, a CD3 x CD123 dual affinity retargeting antibody, which facilitates the recruitment of T cells to CD123^+^ AML cells. Adoptive cell transfer therapies with chimeric antigen receptor (CAR) T and natural killer (NK) cells are currently being developed (5). Nevertheless, re-establishing efficient anti-tumor immunity in a relapsed patient remains a challenge, resulting in dismal clinical outcomes. Why does the transfer of a foreign donor graft achieve a lifelong remission through immunosurveillance in some patients, while in others, the anti-leukemia immune response is inefficient or fails after time? How can donor immune cell activity be reinvigorated to boost the anti-leukemia activity? Is there a way to make tumor cells more susceptible to immune-mediated killing?

Cellular metabolism is now an established hallmark of cancer (8) and an essential determinant of T cell survival, differentiation, and function (9, 10). In this review, we focus on alterations of the metabolic environment as a mechanism of immune escape after allo-HCT. We discuss current knowledge about how immune cell and leukemia cell metabolism regulate the anti-tumor immune response, review recent insights about the role of systemic metabolism, and point out future research directions.

## 2 Graft-versus-leukemia immunity and immune escape after allo-HCT

### 2.1 Biology of the graft-versus-leukemia effect

The term graft-versus-leukemia (GVL) effect refers to the immune response after an allo-HCT that is directed against the malignant cells in the recipient. In this process, donor immune cells eliminate residual leukemic cells to induce long-term remission (11). A potent GVL effect is essential for preventing leukemia relapse (12). However, the GVL effect must be carefully balanced with the risk of graft-versus-host disease (GVHD). GVHD occurs when the alloimmune response targets healthy recipient tissues, causing inflammation in multiple organs, including the gastrointestinal tract, liver, and skin (13). Achieving the proper equilibrium between the GVL effect and GVHD remains a challenge (14). For instance, the depletion of T cells in the donor graft has been shown to prevent GVHD but also increases the risk of relapse by dampening the GVL effect (15). On the other hand, enhancing the GVL effect through DLI is associated with the risk of inducing GVHD (16). These are only two examples of the balancing act between a potent GVL effect needed to prevent leukemia relapse and the risk of GVHD.

For the initiation of the alloimmune response, the T cells first have to recognize the leukemia cells as foreign. This requires the interaction between the T cell receptor (TCR) and specific peptides presented on major histocompatibility (MHC) molecules on the surface of AML cells or antigen-presenting cells (APCs) (17). These peptides can be tumor-specific antigens, tumor-associated antigens, or minor histocompatibility antigens (miHAs) (12). While tumor-specific antigens are encoded by a mutation event and are unique to leukemia cells, tumor-associated antigens are overexpressed in leukemic cells but can also be found on normal cells (18). In contrast, miHAs are MHC-bound peptides that differ between recipient and donor due to single nucleotide polymorphisms and are present on both malignant and non-malignant cells (19). This recognition process is crucial for an effective immune response against the leukemic cells.

Besides antigen recognition, further costimulatory signals, such as the binding to CD28, are needed for the T cells to become activated and undergo clonal expansion (20). Both CD4^+^ and CD8^+^ T cells contribute to the GVL activity via distinct mechanisms (17). CD4^+^ and CD8^+^ T cells have the capacity to eradicate the residual leukemia cells through the secretion of perforin and granzyme or by inducing apoptosis via the fas/fas ligand pathway (21). In GVL mouse models, where mice were injected with the murine leukemia cell line L1210 or P815 two days before bone marrow transplantation, mice receiving BM from FasL-deficient mice survived significantly longer than mice transplanted with BM from perforin-deficient mice, indicating that the perforin pathway is more important for the GVL effect than the fas/fas ligand pathway (22). In addition, CD4^+^ T cells secrete various cytokines, such as interferon-γ (IFN-γ) and tumor necrosis factor (TNF), which can activate other immune cells, promote inflammation, and further amplify the anti-leukemic immune response (23). Blockade of TNF using an anti-TNF antibody massively shortened the survival in the L1210 and P815 mouse leukemia models, demonstrating that TNF plays a critical role in this setting (22).

In addition to T cells, NK cells contribute to the anti-tumor immune response. NK cells are the earliest lymphocytes that recover after allo-HCT and can thus exert a GVL effect even before T cell reconstitution (24). High levels of donor NK cell chimerism early after allo-HCT are associated with a reduced risk of relapse (25). NK cells have the ability to directly kill tumor cells, as they do not require MHC-bound presentation of self-peptides. The key to NK effector function is the balance between the signaling of inhibitory and activating cell surface receptors. Upon activation, NK cells mediate the killing of leukemic cells by the secretion of cytolytic granules and cytokines, such as IFN-γ and TNF (26). Together with T cells, NK cells thereby also contribute to malignancy control.

### 2.2 Mechanisms of relapse after allo-HCT

Malignancy relapse after allo-HCT can be caused by several immune cell-independent and immune-mediated mechanisms (27). Two central immune cell-independent mechanisms are the acquisition of novel oncogenic mutations and the loss of tumor-suppressor genes in tumor cells (28). Genomic analysis of 23 paired AML patient samples collected at diagnosis and at relapse after allo-HCT showed that the leukemic clones at the two time points were genetically different. In detail, 10 patients gained new karyotypic abnormalities, and 13 patients showed changes in the genetic mutation profile, with TET2 and TP53 being the most frequent newly mutated genes (29).

A central immune-mediated mechanism of relapse is the dysregulation of human leukocyte antigen (HLA) expression on AML cells. Here, two modalities have been established in patients. First, in allo-HCT cases where the donor and recipient are not fully HLA-matched, a so-called copy-neutral loss of heterozygosity (CN-LOH) can lead to loss of the mismatched HLA haplotype. This is a genomic event, in which the mismatched HLA haplotype is deleted, while the matched is duplicated. Multiple studies have shown that CN-LOH occurs in up to 30% of patients with relapse after haploidentical stem cell transplantation (30–32). Mismatched HLA loss prevents T cells from recognizing the leukemic cells. In an *in vitro* killing assay, the patient T cells after transplantation as well as the donor T cells were not able to recognize leukemic cells in the case of CN-LOH (30). Importantly, after CN-LOH the overall HLA expression is not reduced, which is why an NK cell response is not activated (33). Furthermore, downregulation of HLA molecules on tumor cells can lead to immune evasion in the case of fully HLA-matched allo-HCT. Two studies analyzing samples from AML patients at the time of diagnosis and relapse after allo-HCT found downregulation of HLA class II molecules on the cell surface of leukemic cells in 39% to 50% of patients (34, 35). Downregulation of HLA class II has also been reported in patients with other hematological malignancies, such as chronic myeloid leukemia (36) and B cell lymphoma (37). The decreased expression of HLA class II impairs the recognition by CD4^+^ T cells and thus reduces the GVL effect (33). Another common immune escape mechanism is the expression of inhibitory immune checkpoint ligands (38). Since the balance of co-stimulatory and co-inhibitory signals influences whether a T cell becomes activated upon the TCR-MHC binding, the upregulation of inhibitory receptors on the leukemic cell surface inhibits T cell activation. A retrospective immunophenotypic analysis of 33 AML patient samples showed an increased expression of the inhibitory molecules PD-L1, B7-H3, and PVRL2 at relapse after allo-HCT compared to diagnosis in up to 40% of the patients (34). Until now, it is poorly understood by which cell-intrinsic mechanisms leukemia cells increase the expression of immune checkpoint ligands. However, there are some studies in other malignancies. For instance, the activation of aberrant janus kinase (JAK) signaling increased PD-L1 expression in Hodgkin lymphoma (39). In myeloproliferative neoplasms, the point mutation JAK^V617F^ drove PD-L1 expression (40). Overall, the downregulation of HLA class II, loss of mismatched HLA, and expression of inhibitory immune checkpoint molecules represent important relapse mechanisms.

Furthermore, tumor cells create an immunosuppressive environment by augmenting the secretion of anti-inflammatory cytokines, such as TGF-ß and IL-4, while reducing the secretion of pro-inflammatory cytokines, such as IL-15 and IFN-γ (27). In a study outside of the allo-HCT setting including 393 acute lymphoblastic leukemia (ALL) patients, low levels of IFN-γ were connected with high-risk B-lineage ALL, suggesting that decreased IFN-γ possibly contributed to escape from immunosurveillance (41). In patients with hematological malignancies who underwent allo-HCT, relapse was associated with low serum levels of IL-15 (42). Moreover, studies in the absence of allo-HCT indicate that leukemic cells might influence the expression of MHC class II molecules by producing TGF-ß and IL-4. TGF-ß-mediated signals were found to be activated in mouse chronic myeloid leukemia-initiating cells *in vitro* (43) and elevated transcript levels of IL-4 were found in human primary chronic myeloid leukemia cells (36). Both TGF-ß and IL-4 are known to antagonize MHC class II expression (44–46) and might contribute to rendering the tumor cells less immunogenic. As proof of principle that local cytokine concentration regulates anti-tumor immunity, a recent study showed that the use of the CD3 x CD123 antibody flotetuzumab or CAR123-directed T cells led to the localized release of IFN-γ from T cells and, subsequently, MHC class II upregulation on AML (47). Further studies are required to gain more knowledge about how local cytokine concentrations can be modulated to achieve optimal anti-tumor immunity.

Metabolites can also contribute to an immunosuppressive environment and support tumor immune evasion. For instance, tumor cells can increase the uptake or catabolism of nutrients essential for immune cells, thus leading to a metabolic competition. Furthermore, tumor cells can secrete metabolites that impair the function of pro-inflammatory immune cells and support the expansion of anti-inflammatory subsets. In addition, metabolic processes in AML cells can regulate the surface expression of inhibitory checkpoint ligands. Finally, systemic metabolism can affect T cell phenotypes and anti-tumor function. In the following sections, we will first focus on AML and T cell metabolism separately before discussing recent evidence of how metabolism influences tumor-immune cell interactions and immune evasion.

## 3 AML metabolism

**Figure 1:**
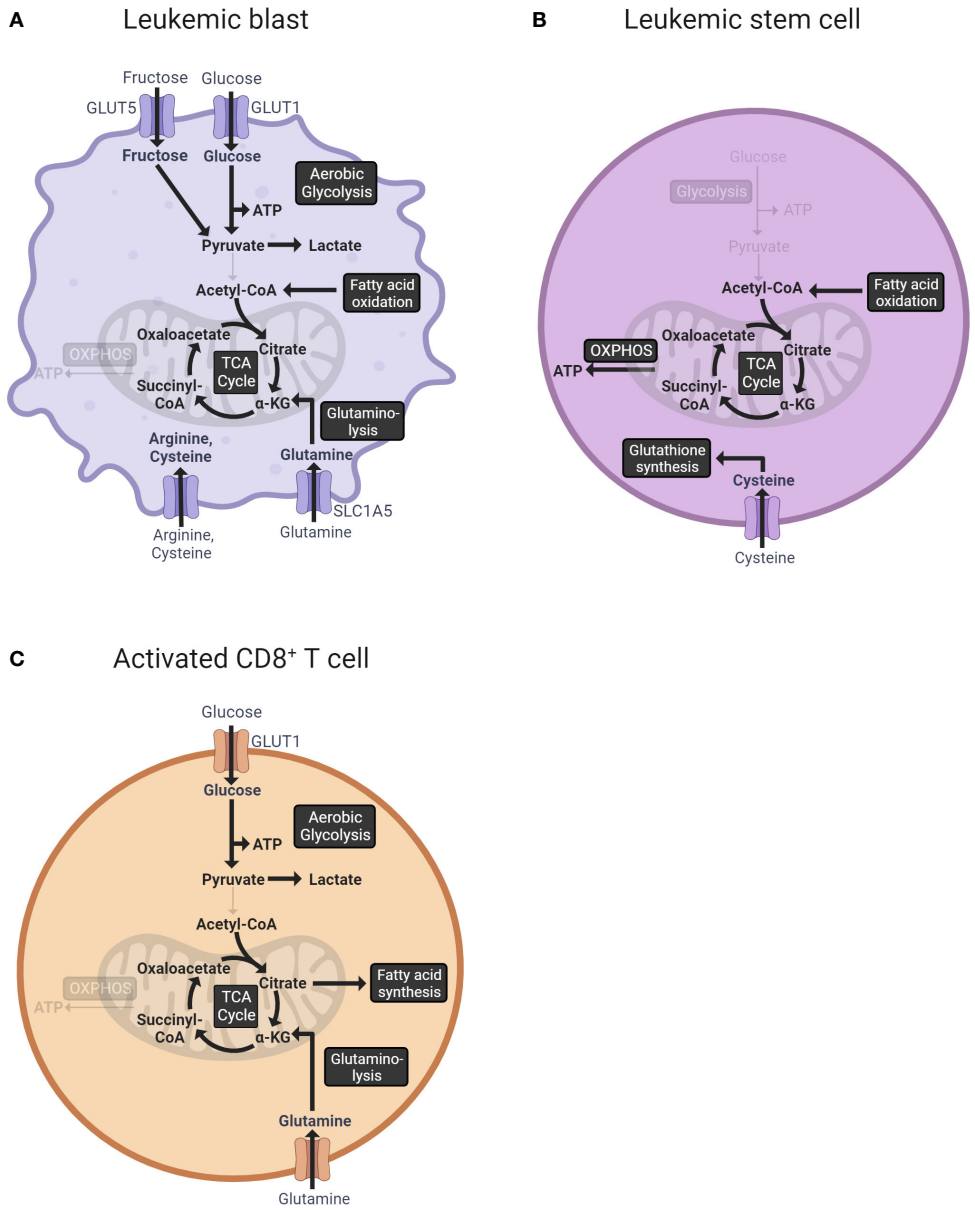
Overview of the central metabolic pathways of leukemic blasts **(A)**, leukemic stem cells **(B)**, and activated T cells **(C)**. **(A)** Leukemic blasts import glucose via the glucose transporter GLUT1 (50). The ingested glucose is metabolized to pyruvate in the process of glycolysis and subsequently fermented to lactate (51). Additionally, leukemic cells import fructose via GLUT5 under low extracellular glucose levels (52). Fatty acids are oxidized to acetyl-CoA, which enters the TCA cycle (53). Glutamine, imported via glutamine transporter SLC1A5, is converted into the TCA cycle intermediate α-ketoglutarate in the process of glutaminolysis (54). Other amino acids, especially arginine and cysteine, are increasingly imported from the extracellular space (55, 56). **(B)** Leukemic stem cells predominantly use OXPHOS for ATP production (57). The TCA cycle is fueled by the oxidation of fatty acids (58, 59). Moreover, LSCs import cysteine, which is used for glutathione synthesis (60). **(C)** Activated CD8^+^ T cells use aerobic glycolysis as the main energy source (61). Fatty acid synthesis is upregulated resulting in an accumulation of fatty acid metabolites needed for membrane synthesis (62). Furthermore, T cells increase the uptake of glutamine, which, converted to α-ketoglutarate, is used to replenish the TCA cycle (63). GLUT1, glucose transporter 1; GLUT5, glucose transporter 5 (mediates fructose uptake); ATP, adenosine triphosphate; CoA, coenzyme A; TCA, tricarboxylic acid; α-KG, α-ketoglutarate; OXPHOS, oxidative phosphorylation; SLC1A5, solute carrier family 1 (neutral amino acid transporter) member 5.

Leukemic cells, characterized by uncontrolled proliferation and impaired differentiation capacity, show extensive metabolic alterations compared to healthy hematopoietic cells. In fact, the deregulation of cellular metabolism is a hallmark of cancer (48). To expand rapidly, leukemic cells have to generate biomass. Thereby, they display a highly diverse and flexible metabolism (49) (**Figure 1A**).

Glucose metabolism plays a central role in generating the biomass required for the rapid expansion of leukemic cells. In an AML mouse model with BCR/ABL and NUP98/HOXA9 fusion proteins, the leukemic cells dramatically increased glucose uptake up to 20-fold compared to normal hematopoietic cells (64). The cellular uptake of glucose is mediated by glucose transporters (GLUT), which are upregulated in many types of cancer (65). Analysis of 108 AML patient samples via qPCR showed that GLUT1 was increased in bone marrow mononuclear cells of AML patients without remission compared to healthy individuals and patients with complete or partial remission (50). Moreover, a study using leukemia cell lines as well as patient samples demonstrated that AML cells additionally upregulated GLUT5, enabling fructose uptake to compensate for glucose deficiency under low extracellular glucose levels (52). The ingested glucose was preferentially fermented to lactate after passing through glycolysis instead of entering the tricarboxylic acid (TCA) cycle, regardless of the presence of oxygen. This shift towards aerobic glycolysis, named Warburg effect (51), enables the cells to produce ATP as well as accumulate precursor molecules and reducing equivalents needed for a rapid cell division (66). In AML patients’ serum, increased amounts of pyruvate and lactate were detected compared to the healthy controls (67). The deletion of pyruvate kinase M2 (PKM2), which catalyzes the rate-limiting conversion from phosphoenolpyruvate to pyruvate in glycolysis, in mouse BM cells carrying BCR-ABL or MLL-AF9 rearrangements reduced leukemia cell growth *in vivo* without perturbing HSCs (68). Overall, aerobic glycolysis is one of the essential pathways for leukemic cells.

Besides glucose, leukemia cells also increase the uptake and consumption of amino acids, most importantly glutamine (69). In the process of glutaminolysis, glutamine is converted into α-ketoglutarate, which can be used to replenish the TCA cycle (70). While the glutamine plasma concentration in healthy persons amounts to 0.6 nM to 0.8 nM, the glutamine concentrations measured in the serum of AML patients were below 0.3 nM (71, 72). Glutamine is mainly imported via the high-affinity glutamine transporter SLC1A5. A shRNA-mediated knockdown of SLC1A5 induced apoptosis in the AML cell lines MOLM-14 and HL-60 and reduced the growth of MOLM-14 tumors in a xenograft mouse model underlining the importance of glutamine for leukemia cell metabolism (54). Importantly, a genome-wide CRISPR-Cas9 knockout screen identified the enzyme glutaminase (GLS) as a major metabolic vulnerability in FLT3-ITD-mutated AML cells treated with FLT3 tyrosine kinase inhibitors (73). Genetic or pharmacological silencing of GLS function synergized with the FLT3 inhibitor AC220 to induce apoptosis in the FLT3-ITD-mutated human cell lines MV-4-11 and MOLM13 (73). Moreover, glutamine is an important metabolite for the synthesis of the reactive oxygen species (ROS) scavenger glutathione. Blocking glutamine metabolism with the glutaminase inhibitor CB-839 significantly impaired glutathione production in multiple AML cell lines, leading to the accumulation of mitochondrial ROS and apoptosis (73, 74).

Apart from glutamine, arginine has been proven to be important for AML cells (55, 75). Arginine is a semi-essential amino acid and is crucial to providing amine groups and aspartate for the production of nucleotides (76). The majority of leukemic cells do not express argininosuccinate synthetase-1 (ASS1), which is needed for the intracellular synthesis of arginine and thus depend on arginine uptake (55). In line with these findings, the arginine transporters CAT-1 and CAT-2B were found to be constitutively expressed on AML blasts, and the plasma arginine levels of AML patients were significantly reduced compared to healthy individuals (75). Moreover, the treatment with a human recombinant arginase (BCT-100), which catabolizes the cleavage of arginine and thus reduces its availability for uptake, inhibited the proliferation of primary AML blasts *in vitro* (75). Nevertheless, the clinical implementation of treatments targeting arginine metabolism has so far been challenging. A randomized clinical trial testing low-dose cytarabine in combination with the human recombinant arginase BCT-100 versus low-dose cytarabine alone in 86 AML patients over 60 years of age found no difference in the overall and median survival between the two groups (77). Another approach is to inhibit arginase together with inducible nitric oxide synthase (iNOS). In *in vitro* experiments, the combination of the small molecule inhibitor of arginase NOHA and the iNOS inhibitor L-NMMA enhanced the proliferation and cytotoxicity of T cells and CAR T cells (78, 79). Moreover, treatment with NOHA and L-NMMA restored the proliferation of HSCs and reduced the expression of CD206 on monocytes (78).

Furthermore, AML cells depend on the uptake of extracellular cysteine due to their inability to generate cysteine from methionine. Cysteine depletion in the cell culture medium decreased the viability and proliferation of primary AML cells and most AML cell lines. Since cysteine is an important precursor for the synthesis of glutathione, depletion resulted in increased ROS levels, which led to cell death via ferroptosis. While therapeutic targeting of cysteine uptake alone using the cysteine/glutamate antiporter inhibitor sulfasalazine showed limited efficacy in AML cell lines, combination with the γ-glutamylcysteine synthase (GCL) inhibitor L-Buthionine sulfoximine, which inhibits the production of glutathione, reduced proliferation in multiple AML cell lines and primary AML samples (56). Taken together, amino acids, specifically glutamine, arginine, and cysteine, play an essential role in the metabolism of leukemic cells.

In addition, leukemia cells undergo changes in lipid metabolism (80). Profiling of the lipid composition in five AML cell lines revealed significant differences in the expression of lipid species between the cell lines at steady state. However, under global serum deprivation, the cell lines showed variations in the same lipid species, suggesting a shared adaptive mechanism to nutrient stress. More precisely, phosphatidyl-serine, phosphatidyl-ethanolamine, and phosphatidyl-choline were decreased. Lipid profiling of plasma samples from AML patients revealed an incresed concentration of phosphatidyl-inositol and free fatty acids in the plasma of patients with an unfavorable prognosis (n=5) compared to patients with a favorable prognosis (n=5) (81). Another study analyzing the serum lipid levels in 214 AML patient samples found, among others, apolipoprotein B and high-density lipoprotein cholesterol to be prognostic factors for the clinical outcome (82). Furthermore, AML blasts can induce the phosphorylation of hormone-sensitive lipase in bone marrow adipocytes and activate lipolysis, leading to the release of fatty acids from adipocytes (83). The absorbed fatty acids can be oxidized to acetyl-CoA, thus serving as an alternative fuel for the TCA cycle and OXPHOS (53). Analysis of public microarray datasets revealed an increased expression of the carnitine palmitoyltransferase 1A (CPT1A), which catalyzes the first step of fatty acid oxidation, in the bone marrow, peripheral blood, and CD34^+^ cells of AML patients compared to healthy individuals. Moreover, a high expression of CPT1A was associated with a shorter survival (84). Likewise, the carnitine transporter CT2 was found to be highly expressed in AML cell lines and primary AML cells. An shRNA-mediated knockdown of CT2 in the AML cell lines OCI-AML2 and HL-60 decreased the viability and growth (85). In summary, leukemia cells exhibit modulated lipid metabolism that favors their rapid growth.

AML is a heterogeneous disease group characterized by a variety of chromosomal and cytogenetic aberrations, and it is conceivable that different AML subtypes might be characterized by distinct metabolic programs. Many genetic alterations lead to constitutive PI3K-AKT-TOR pathway activation, enhancing glucose uptake, glycolytic flux, amino acid uptake, acetyl-CoA production, and synthesis of lipids and nucleotides (86). Additionally, several studies investigated the connection between specific mutations and distinct metabolic abnormalities. For example, internal tandem duplication (ITD) mutation in FLT3 has been shown to upregulate mitochondrial hexokinase and increase the dependency on aerobic glycolysis in murine and human AML cell lines (87). Additionally, FLT3-ITD^+^ primary human AML cells had a lower abundance of ceramides compared to samples from FLT3-ITD^-^ patients. Mechanistically, FLT3 inhibition with sorafenib, crenolanib, or AC220 restored the ceramide abundance in MV-4-11 and MOLM-14 cell lines, and the accumulated mitochondrial ceramides facilitated mitophagy leading to cell death (88). FLT3-mutant AML was also shown to be particularly susceptible to ferroptosis, an iron-dependent form of cell death. Inhibition of FLT3 or the transcription factor C/EBPα downregulated the expression of stearyl-CoA-desaturase leading to impaired incorporation of fatty acids into lipids and elevated susceptibility to lipid redox stress (89). Moreover, metabolomics of AML patient samples revealed increased serum levels of choline, trimethylamine N-oxide, and leucine in patients carrying an NPM1 mutation together with mutations in cohesion complex and DNA damage response (90). Furthermore, somatic mutations in isocitrate dehydrogenase 1 and 2 (IDH1 and IDH2) lead to a neomorphic enzyme activity. Instead of catalyzing the oxidative decarboxylation of isocitrate to α-ketoglutarate, mutant IDH catalyzes the reduction of α-ketoglutarate into R-2-Hydroxyglutarate (R-2-HG) (91, 92). Overall, the knowledge about the relationship between genomic mutations and AML metabolism is limited to date, and further research is needed to explore the metabolic dependencies associated with distinct genetic profiles.

Notably, the metabolism of leukemic stem cells (LSCs) is distinct from that of rapidly proliferating leukemia blasts (93, 94). LSCs are a subpopulation of AML cells that reside in the bone marrow niche and have a quiescent cell cycle status and self-renewal properties (95). Moreover, LSCs are often resistant to chemotherapy and are thought to drive relapse (96, 97). In contrast to leukemic blasts, LSCs predominantly use OXPHOS and not glycolysis for ATP production (57). Since almost no pyruvate is available due to low glycolytic activity, LSCs require amino acids and/or fatty acids to fuel the TCA cycle and OXPHOS (98, 99). Similar to bulk leukemic cells, the amino acid cysteine has been found to be particularly important for the survival of LSC. In human LSCs, the depletion of cysteine *in vitro* led to impaired glutathione synthesis and a reduced glutathionylation of succinate dehydrogenase A, the key component of electron transport chain complex II. The resulting inhibition of OXPHOS led to LSC death (60). The specific metabolism of LSCs represents an opportunity for therapeutic targeting (58, 59). However, while *de novo* LSCs are described as metabolically inflexible, relapsed/refractory LSCs display a higher metabolic plasticity (99). For instance, the withdrawal of amino acids led to the cell death of LSCs isolated from *de novo* AML patients, whereas LSCs from relapsed AML patients were not dependent on amino acids because of their ability to balance energy requirements through fatty acid metabolism (98).

While chemotherapy eliminates most bulk leukemic cells, it is rarely effective in eliminating LSCs (58). A growing body of recent work has focused on identifying unique metabolic features in therapy-resistant LSCs (100) (**Figure 1B**). The combination treatment of the BCL2 inhibitor venetoclax and the hypomethylating agent azacytidine has shown significantly better clinical outcomes than conventional treatment in newly diagnosed elderly AML patients (101, 102). Further analysis demonstrated that in patients treated with venetoclax and azacytidine, the LSC population was decreased due to disruption of LSC energy metabolism (98, 101). More precisely, the combination of venetoclax and azacytidine reduced the uptake of amino acids, resulting in a decreased OXPHOS (98). Since LSCs depend on OXPHOS (57, 103), disruption of this process causes selective cell death of LSCs (98, 101). Relapse/refractory LSCs, however, exhibit a higher metabolic plasticity (99). As mentioned above, LSCs isolated from relapsed AML patients were able to compensate for the amino acid deprivation through upregulation of fatty acid metabolism (98). These LSCs could be re-sensitized by combining venetoclax/azacytidine with inhibitors of fatty acid transport, such as etomoxir and sorbitan sesquioleate (98, 104). The combination of etomoxir with venetoclax/azacytidine reduced OXPHOS in human LSCs ex vivo, leading to a lower viability as well as a decreased tumor burden in a xenograft mouse model with primary AML samples (104). Treatment of LSCs from relapsed AML patients with sorbitan sesquioleate and venetoclax/azacytidine also significantly decreased OXPHOS and reduced viability (98). Moreover, metabolomics profiling of six *de novo* AML patients and six patients with relapsed AML after induction therapy revealed enhanced nicotinamide levels and increased nicotinamide metabolism in relapsed LSCs. The enhanced nicotinamide metabolism resulted in increased total energy metabolism, thus bypassing the cytotoxic effect of venetoclax/azacytidine therapy in relapsed/refractory LSCs (105). Collectively, these examples demonstrate the importance of LSC metabolism in therapy resistance.

## 4 T cell metabolism

The metabolism of T cells is intricately linked to their cell function, and distinct metabolic programs are required at each stage of activation and differentiation. Naive CD8^+^ T cells, which remain in a quiescent state, fulfill their relatively low metabolic requirements primarily by pyruvate and fatty acid oxidation via the TCA cycle and subsequent OXPHOS (106). Upon antigen recognition, CD8^+^ T cells expand clonally, for which both a sufficient energy supply and a large number of biomolecules are needed. Consequently, the transition from resting naive T cells to activated T cells requires substantial metabolic reprogramming (107), which we will review briefly here.

A characteristic of activated CD8^+^ T cells is a switch towards aerobic glycolysis as the main energy source, commonly referred to as the Warburg effect (61). In this process, the pyruvate produced from glucose during glycolysis is fermented to lactate despite the availability of oxygen. Although aerobic glycolysis yields only two ATP molecules per glucose molecule, in contrast to up to 36 ATP molecules generated by OXPHOS, it offers the advantage of providing metabolic intermediates crucial for cell growth and proliferation (107). Moreover, it induces the pentose phosphate pathway, which supplies the cells with ribose 5-phosphate, a building block for nucleic acid synthesis, and NADPH (108). NADPH is required for catabolic pathways, such as the synthesis of fatty acids and amino acids, and the maintenance of the antioxidant glutathione (109). The neutralization of ROS is particularly important because activated T cells experience high levels of oxidative stress due to the increased non-mitochondrial oxygen consumption rate (110). Furthermore, CD8^+^ T cells increase glutamine uptake. In the process of glutaminolysis, glutamine is converted into α-ketoglutarate, which can subsequently enter the TCA cycle. Additionally, glutaminolysis provides NADPH to support lipid and nucleotide biosynthesis and the maintenance of glutathione (63). Apart from glucose and glutamine, lipids are an effective energy source as well as biosynthetic intermediates. Activated CD8^+^ T cells upregulate fatty acid synthase while decreasing fatty acid oxidation, leading to the accumulation of fatty acid metabolites needed for membrane synthesis (62). Furthermore, complex lipids support effector T cell signaling and function. Activated CD8^+^ T cells accumulate saturated phosphatidylinositides (PIPn) as opposed to naive CD8^+^ T cells, which are abundant in polyunsaturated PIPn. Saturated PIPn act as superior substrates for the enzymes PIP5K and PLC for the generation of second messengers and thus support downstream signaling essential for survival and cytokine production (111). In summary, CD8^+^ T cells exhibit metabolic changes upon activation, favoring aerobic glycolysis, glutaminolysis, and lipid accumulation to support proliferation and T cell functions (**Figure 1C**).

After antigen elimination, a small subset of cells differentiates into long-lasting memory T cells, which provide a fast and robust immune response upon re-exposure to the antigen. As a quiescent cell population, memory T cells have a catabolic metabolism and rely on OXPHOS fueled mainly by fatty acid oxidation (112–114).

Moreover, the metabolism of T cells also varies depending on their lineage commitment. The proinflammatory CD4^+^ T cell lineages Th1, Th2, and Th17 mainly use glycolysis to cover their energy needs (115). In contrast, CD4^+^ regulatory T cells (T_regs_), which dampen the immune response and promote tolerance, are less reliant on glycolysis and instead use mitochondrial metabolism and OXPHOS for energy production (116). Due to the limited reliance on glycolysis, T_regs_ may have a metabolic advantage to survive and function in glucose-depleted conditions (117, 118). Notably, fatty acid metabolism is essential for T_reg_ generation. Blockade of fatty acid oxidation using the carnitine palmitoyl-transferase-1 inhibitor etomoxir suppressed the differentiation of mouse CD4^+^ T cells into T_regs_ *in vitro* (115). These distinct metabolic requirements are critical for the optimal function of different T cell subsets and result in distinct fates in the metabolically altered tumor microenvironment.

## 5 Metabolic regulation of the GVL immunity

An increasing body of evidence suggests that the strength of T cell anti-leukemia responses is regulated by metabolic cues, both within localized interactions between AML cells and immune cells, and on the organismal level. In the following sections, we will first discuss how altered AML metabolism affects immune function by shaping the microenvironment. Next, we will review the literature on how metabolism regulates the expression of inhibitory checkpoint ligands on the AML cell surface. We will then address the potential impact of myeloid cell metabolism on the anti-tumor response. Finally, we will focus on the metabolic regulation of immune responses on the organismal level. In each section, we will additionally highlight ways in which the metabolism could be therapeutically modulated to enhance the GVL effect.

### 5.1 AML metabolism generates an immunosuppressive metabolic microenvironment

#### 5.1.1 Arginase II

**Figure 2:**
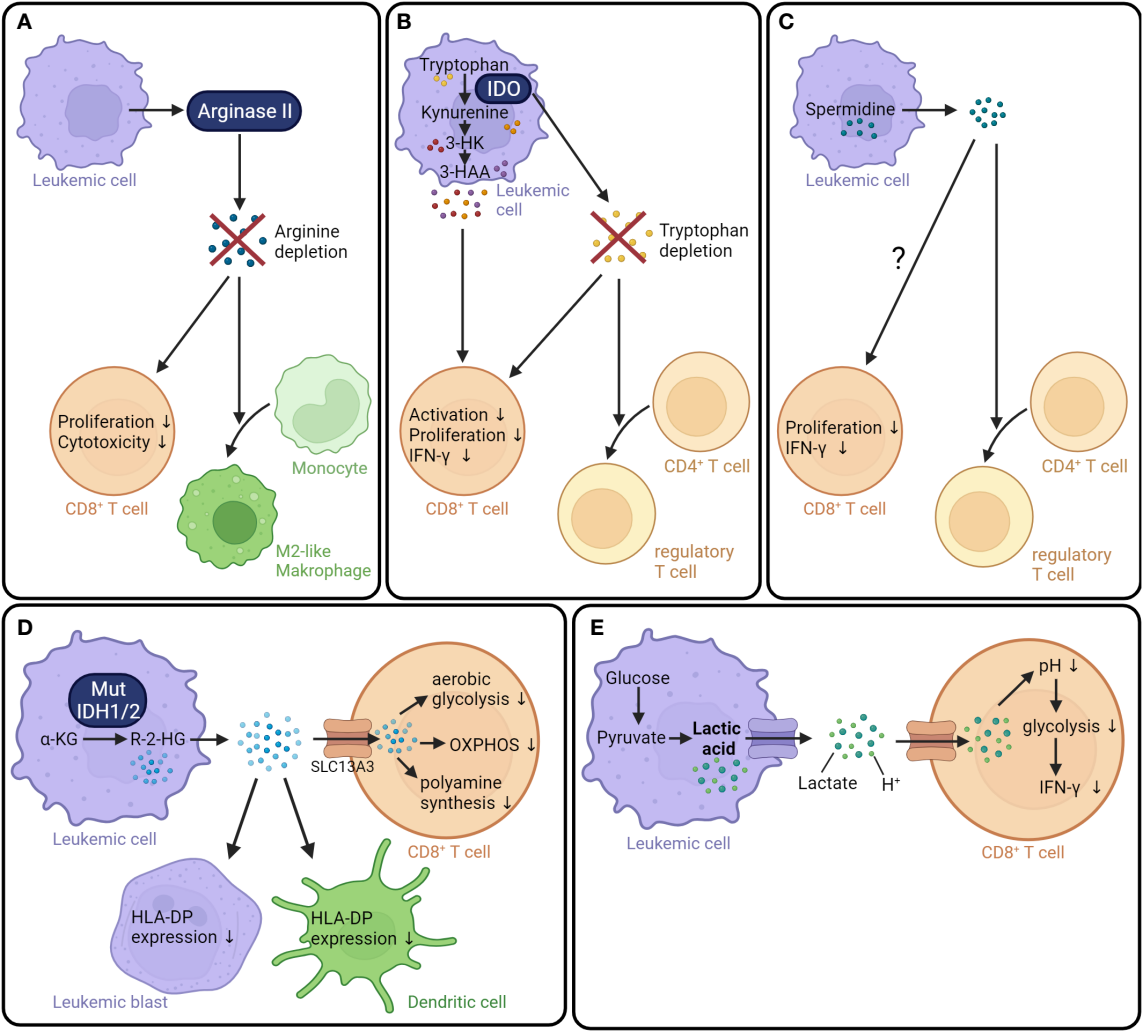
AML metabolism generates an immunosuppressive microenvironment. **(A)** Leukemic cells secrete arginase II, which leads to the depletion of arginine in the tumor microenvironment resulting in decreased T cell proliferation and cytotoxicity. Moreover, surrounding monocytes are polarized into tumor-supporting M2-like phenotype (78, 79). **(B)** IDO catalyzes the degradation of tryptophan into kynurenine resulting in a tryptophan depletion in the tumor microenvironment. The absence of tryptophan as well as elevated levels of kynurenine derivates, namely 3-hydroxykyrunenine and 3-hydroxyanthranilic acid, decrease T cell activation, proliferation, and IFN-γ production (120–123). In addition, tryptophan depletion promotes the conversion of CD4^+^ T cells into T_regs_. **(C)** The polyamine spermidine produced by leukemic cells boosts the differentiation of CD4^+^ T cells into T_regs_ (124). Additionally, spermidine might negatively influence the CD8^+^ T cell proliferation and function (125). **(D)** Mutant IDH1/2 converts the TCA cycle intermediate α-ketoglutarate into the oncometabolite R-2-HG (92, 93). R-2-HG is released into the extracellular space (126) and taken up by CD8^+^ T cells via the transporter SLC13A3 (127). In CD8^+^ T cells, R-2-HG suppresses aerobic glycolysis, OXPHOS, and polyamine synthesis (127, 128). Furthermore, R-2-HG reduces the HLA-DP expression on leukemic blasts and dendritic cells (129). **(E)** Lactic acid is a product of aerobic glycolysis. At physiological conditions, lactic acid is dissociated into lactate and a proton. In CD8^+^ T cells, lactic acid reduces the intracellular pH, resulting in decreased glycolysis and IFN-γ production (130). IDO, Indoleamine-2,3-dioxygenase; 3-HK, 3-hydroxykyrunenine; 3-HAA, 3-hydroxyanthranilic acid; IFN-γ, interferon γ; IDH1/2, isocitrate dehydrogenase 1/2; α-KG, α-ketoglutarate; R-2-HG, R-2-hydroxyglutarate; SLC13A3, Solute carrier family 13 member 3; OXPHOS, oxidative phosphorylation; H^+^, proton.

Leukemic cells can modulate the anti-tumor response by depleting nutrients in the tumor microenvironment, either through direct uptake or through the activity of catabolic enzymes. One example is the depletion of the amino acid arginine. Activated T cells dramatically increase their uptake and catabolism of arginine to support central metabolic processes (119). Several studies have shown that AML cells decrease extracellular arginine availability by secreting arginase II, an enzyme that catalyzes the hydrolysis of arginine into ornithine and urea (78, 79). Analysis of 15 AML patients found that arginase II was expressed and released by leukemic blasts. Cultivation in plasma obtained from AML patients significantly inhibited the proliferation of T cells and hematopoietic progenitor cells. These effects were reversed by the addition of inhibitors of arginase and iNOS. Moreover, arginase II contributed to the ability of leukemic blasts to polarize the surrounding monocytes into an immunosuppressive “M2-like” phenotype (78). Another study with 80 AML patients found that the plasma arginine concentration was significantly lower compared to healthy controls. In line with this observation, the administration of recombinant arginase in NOG-SCID mice engrafted with human lymphocytes reduced the serum arginine concentration and interfered with T cell expansion. Moreover, chimeric antigen receptor (CAR) T cells cultivated in low arginine concentrations proliferated less and showed a reduced ability to kill the tumor cell line K562 *in vitro* compared to CAR T cells grown in a standard medium (79). Overall, these findings indicate that AML-derived arginase II can lead to the depletion of arginine in the tumor microenvironment, and this in turn might impair T cell proliferation and function (**Figure 2A**).

#### 5.1.2 Indoleamine-2,3-dioxygenase

Another well-studied example is the depletion of tryptophan by indoleamine-2,3-dioxygenase (IDO). IDO catalyzes the initial rate-limiting step of tryptophan degradation, the oxidation of tryptophan into N-formylkynurenine (131). IDO is expressed by a variety of human cell types, including macrophages and dendritic cells, and its expression is regulated by IFN-γ (132, 133). Analysis of primary AML cells from 76 patients at the time point of diagnosis found that IDO protein was constitutively expressed in the AML cells of around 50% of the patients. In contrast, IDO expression was not detectable in normal hematopoietic BM cells (134). A study investigating the role of IDO in newly diagnosed childhood AML did not detect a constitutive IDO expression on AML blasts. However, IDO expression was upregulated upon treatment with IFN-γ in 19 out of 37 samples and was associated with worse survival (135). IDO is known to play an important role in immune tolerance by regulating NK cells, T cells, and myeloid-derived suppressor cells (136). Co-culture experiments revealed that IDO-expressing AML blasts reduced the ability of both CD4^+^ and CD8^+^ T cells to produce IFN-γ (135). Over the past decades, three mechanisms through which IDO exerts immunosuppressive effects have emerged. First, a shortage of tryptophan due to IDO consumption can inhibit T cell activation (120, 137). Tryptophan is an essential amino acid crucial for cell survival and protein biosynthesis (138). Cultivation of T cells in tryptophan-depleted cell culture medium inhibited T cell proliferation and induced apoptosis (137). Second, products of tryptophan catabolism, namely kynurenine derivatives, negatively regulate T-cell proliferation and survival (121, 139). For instance, kynurenine, 3-hydroxykynurenine, and 3-hydroxyanthranilic acid strongly suppressed the response of allogeneic T cells *in vitro* (121). Third, IDO impairs anti-tumor immunity by driving the differentiation of CD4^+^ T cells into immunosuppressive T_regs_ (122, 123, 135). Co-culture of human primary IDO^+^ AML cells led to a conversion of CD4^+^ CD25^-^ into CD4^+^ CD25^+^ T cells. Moreover, intrasplenic injection of IDO^+^ leukemia cells led to an expansion of T_regs_ *in vivo* (122). Taken together, IDO can be expressed by leukemic cells and helps create an immunosuppressive microenvironment by depleting tryptophan, producing immunosuppressive metabolites of the kynurenine pathway, and increasing the number of T_regs_ (**Figure 2B**).

#### 5.1.3 Polyamines

Polyamine metabolism is upregulated in multiple types of cancer (140–142) and plays a role in the regulation of the antitumor immune response (143, 144). In AML, an analysis of the polyamine concentrations in PBMCs collected from 28 patients with newly diagnosed AML revealed that levels of spermidine and spermine were significantly higher in AML patients compared to healthy controls. In line with this observation, the activity of spermidine/spermine N^1^-acetyltransferase (SSAT) was increased. Moreover, high SSAT activity was associated with an increased white blood cell count (145). In another study, the serum total polyamine concentration in patients with AML (n=13), Hodgkins´s disease (n=55), and non-Hodgkin lymphoma (n=21) was examined. An increased polyamine concentration compared to healthy controls (n=8) was measured in all three types of hematological tumors (146). The higher concentrations of polyamines might affect the activity of CD8^+^ T cells; however, it is not yet clear in which direction as studies concerning AML and allo-HCT specifically have not been published. While some reports suggest that spermidine suppresses T cell function (125, 143, 144, 147), others indicate that spermidine can enhance T cell function (148, 149). One study demonstrated, for example, that the addition of spermidine suppressed the proliferation of CD8^+^ T cells activated with anti-CD3 and anti-CD28 antibodies *in vitro* as well as *in vivo*. Mechanistically, spermidine downregulated cholesterol levels in the plasma membrane leading to suppressed TCR clustering. The treatment of B16F10 tumor-bearing mice with the polyamine synthesis inhibitor eflornithine reduced tumor growth and enhanced CD8^+^ T cell infiltration. The combination of eflornithine with an anti-PD-1 antibody further potentiated anti-tumor immunity (125). In contrast, spermidine supplementation was shown to improve the anti-tumor activity of PD-L1 antibody treatment in a MC38 colon cancer model. The addition of spermidine during *in vitro* activation of mouse CD8^+^ T cells resulted in enhanced cytotoxicity. Moreover, spermidine increased the activity of FAO through direct binding to the mitochondrial trifunctional protein, which is a multienzyme complex central for ß-oxidation (148). These data are derived from preclinical models of solid tumors and the influence of polyamines on CD8^+^ T cells in leukemia needs to be specifically investigated in the future.

In addition, the increased spermidine levels in AML patients could possibly influence the GVL effect by disturbing the differentiation of CD4^+^ T cells. When spermidine was added to naïve mouse CD4^+^ T cells cultured under T_H_17 cell-polarizing conditions, the production of IL-17 was reduced and the percentage of Foxp3^+^ cells was increased compared to cells differentiated in the absence of spermidine (124). A higher proportion of T_regs_ due to higher spermidine concentrations could contribute to the immunosuppressive tumor microenvironment in AML (**Figure 2C**).

Of note, polyamine metabolism has been explored as a therapeutic target in AML. Treatment with the polyamine synthesis inhibitor methylglyoxal (bis)guanylhydrazone in combination with the ornithine decarboxylase inhibitor eflornithine has been proven to have anti-leukemia effects in a clinical trial, but showed a high toxicity (150). Moreover, polyamine conjugation represents a way of delivering drugs to tumor cells with high polyamine import. For instance, F14512, a topoisomerase II inhibitor conjugated with a spermine derivative, was developed for the treatment of AML (151).

#### 5.1.4 R-2-Hydroxyglutarate

Another metabolite that has been reported to impair T cell activity is R-2-hydroxyglutarate (R-2-HG) (127, 152). R-2-HG, which is structurally similar to α-ketoglutarate, is produced in AML with mutant isocitrate dehydrogenase 1 and 2 (IDH1 and IDH2). Somatic mutations in IDH1 and IDH2 occur in 6-16% and 8-19% of adult patients with AML, respectively (153). Mutant IDH acquires a neomorphic enzyme activity for the NADPH-dependent reduction of α-ketoglutarate into R-2-HG (91, 92). In leukemic cells from AML patients carrying an IDH1/2 mutation (n=16), the levels of total 2-HG were approx. 50-fold higher compared to cells from AML patients with wild-type IDH1/2 (n=10). Interestingly, the total 2-HG was not only increased intracellularly but also in the serum of patients with an IDH mutation (126), indicating that R-2-HG is released into extracellular space. The released R-2-HG can be taken up by T cells in a paracrine fashion via specific sodium-dependent transporters (127). *In vitro* experiments with mouse and human T cells showed that R-2-HG reduced the proliferation of T cells in a concentration-dependent way (127, 128). Moreover, R-2-HG impaired T cell function by blocking the formation and release of cytotoxic granola as well as IFN-γ production and secretion. Mechanistic studies showed that R-2-HG inhibited lactate dehydrogenase (LDH) activity in mouse CD8^+^ T cells *in vitro*. The blocked aerobic glycolysis forced T cells to produce ATP via OXPHOS, resulting in higher ROS production (128). *In vitro* experiments using human T cells demonstrated that R-2-HG suppressed ATP synthase, leading to reduced oxidative ATP production and attenuated PLC-γ phosphorylation, and consequently, decreased nuclear translocation of the nuclear factor of activated T cells (NFAT). Additionally, R-2-HG inhibited polyamine biosynthesis in human T cells (127). Interestingly, a recent study showed that R-2-HG exposure *in vitro* reduced the HLA-DP expression on antigen-presenting cells and leukemic blasts, resulting in significantly impaired HLA-DP-mediated specific lysis of IDH-mutant AML blasts by TCR-engineered T cells (129). Taken together, R-2-HG produced by mutant IDH interferes with anti-tumor immunity by both hampering T cell activity and decreasing HLA expression on AML cells (**Figure 2D**).

#### 5.1.5 Lactic acid

Another immunosuppressive metabolite secreted by leukemic cells is lactic acid (130). Lactic acid is the product of aerobic glycolysis. As mentioned above, leukemic cells import high amounts of glucose, which is converted to pyruvate in the process of glycolysis and subsequently fermented to lactate despite the presence of oxygen (51). At physiological conditions, lactic acid is dissociated into lactate and a proton (H^+^). Both are exported via monocarboxylate transporters (MCTs), resulting in lactate accumulation and acidification of the extracellular milieu (154).

Interestingly, lactic acid was described as a metabolite that facilitated AML relapse particularly in the post-allo-HCT setting, as it was specifically increased in the serum of AML patients with relapse after allo-HCT (n=7) compared to a time point at which the same patients were in remission. AML cell-derived lactic acid reduced the intracellular pH in T cells, resulting in a decreased transcription of enzymes involved in glycolysis, lower activity of fundamental metabolic pathways, and impaired function (130) (**Figure 2E**). Earlier studies showed that lactic acid was increased in the serum of patients with solid tumors and that exposure to high concentrations of lactic acid severely decreased the proliferation, activation, cytokine production, and cytotoxic function of human cytotoxic lymphocytes *in vitro* (155). Of note, lactic acid provides metabolic support for T_regs_ (156). In contrast to effector T cells, T_regs_ do not only have an advantage in the glucose-depleted tumor microenvironment due to their low glycolytic activity (116), but can also use lactic acid to fuel the TCA cycle. Intratumoral T_regs_ are potentially indeed reliant on lactic acid, as the depletion of the lactic acid transporter MCT1 in mouse T_regs_ reduced their proliferation and suppressive function *in vitro* (156).

Sodium bicarbonate (NaBi) is clinically used to antagonize metabolic acidosis and has already been explored as an experimental treatment for metastasis (157, 158). In an allo-HCT setting, *in vitro* NaBi treatment of murine T cells completely reversed the AML-induced inhibition of T cell glycolytic activity and rescued cell proliferation. Moreover, NaBi enabled T cells to use extracellular LA as an additional fuel source for energy production. In leukemia-bearing mice treated with NaBi, the survival was significantly improved compared to the vehicle group. In addition, CD8^+^ T cells isolated from AML patients treated with NaBi showed increased respiration and production of IFN-γ and TNF. In summary, reversing T cell acidosis by administering NaBi could potentially augment anti-leukemia immune responses (130).

### 5.2 AML metabolism drives inhibitory checkpoint ligand expression

#### 5.2.1 Lactate

Lactate is the lactic acid conjugate formed by the dissociation of a proton. Lactate accumulates within AML cells as a consequence of elevated aerobic glycolysis (51). Constitutive STAT5 activation in human HL-60 AML cells *in vitro* boosted glycolysis and increased the intracellular lactate concentration. Lactate accumulation promoted the nuclear translocation of E3BP and enhanced histone lactylation in the promoter regions of PD-L1 thus elevating PD-L1 transcription. Co-culture with STAT5 constitutively activated AML cells led to a decreased activation of Jurkat T cells, as demonstrated by reduced CD69 expression. This effect was reversed by PD-L1 knock-down or treatment with PD-1 neutralizing antibody. Taken together, lactate accumulation induces PD-L1 upregulation, which might suppress T cell activation (159).

#### 5.2.2 Electron transport chain

OXPHOS is an essential metabolic process for LSC survival and therapy resistance (57, 103). However, OXPHOS can also regulate immune checkpoint expression on AML cells. Blocking the electron transport chain complex I with the selective inhibitor EVT-701 decreased the expression of CD39 and PD-L1 in THP1, MOLM-13, C1498, and L1210 AML cells *in vitro* (160). The electron transport chain may additionally regulate the expression of MHC class I. Knock-out of the electron transport chain complex II in a melanoma mouse model increased the immunogenicity of the tumor cells by enhancing the transcription of MHC class I and other genes involved in antigen presentation and processing, leading to reduced tumor growth (161). Whether this regulation is active in AML, is currently unknown. In summary, the activity of the electron transport chain could possibly influence the immunogenicity of leukemic cells by regulating the expression of MHC and inhibitory immune checkpoint ligands.

#### 5.2.3 Fatty acid synthase

Rapidly dividing AML cells require the synthesis of new fatty acids, especially for membrane formation and the generation of signaling molecules (49). *In vitro* studies with the T cell acute lymphoblastic leukemia cell line Jurkat indicated that fatty acid synthase (FASN) regulates the expression of PD-L1, as inhibiting FASN with orlistat led to a downregulation of PD-L1 (162). FASN was previously shown to be highly expressed in the human AML cell line HL-60 (163) and mRNA levels were significantly higher in AML patient blasts (n=204) compared to HSC from healthy individuals (n=6) (164). Thus, it is possible that the strong FASN expression in AML cells leads to an increased PD-L1 expression.

**Figure 3:**
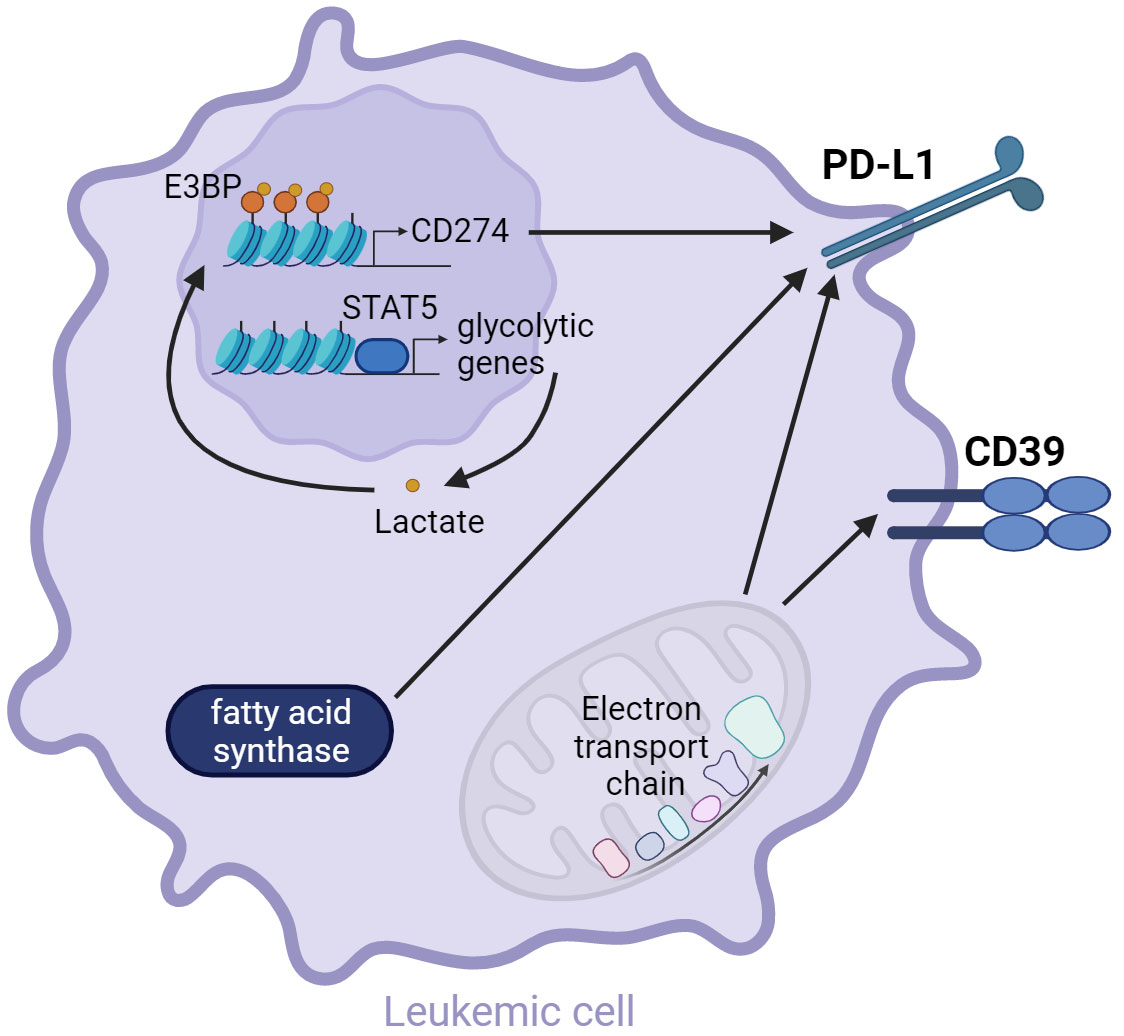
AML metabolism drives inhibitory checkpoint ligand expression. Constitutive STAT5 activation results in the transcription of glycolytic genes. Enhanced aerobic glycolysis leads to the accumulation of lactate in the leukemic cell, promoting the nuclear translocation of E3BP and histone lactylation, which induces the transcription of *CD274*, encoding for PD-L1 (159). Moreover, fatty acid synthase and OXPHOS were shown to regulate PD-L1 expression (160, 162). OXPHOS additionally regulates CD39 expression (160).

The metabolic mechanisms that regulate inhibitory checkpoint ligand expression are summarized in **Figure 3**.

### 5.3 Myeloid cell metabolism influences GVL effect

#### 5.3.1 ATP/Adenosine metabolism

**Figure 4:**
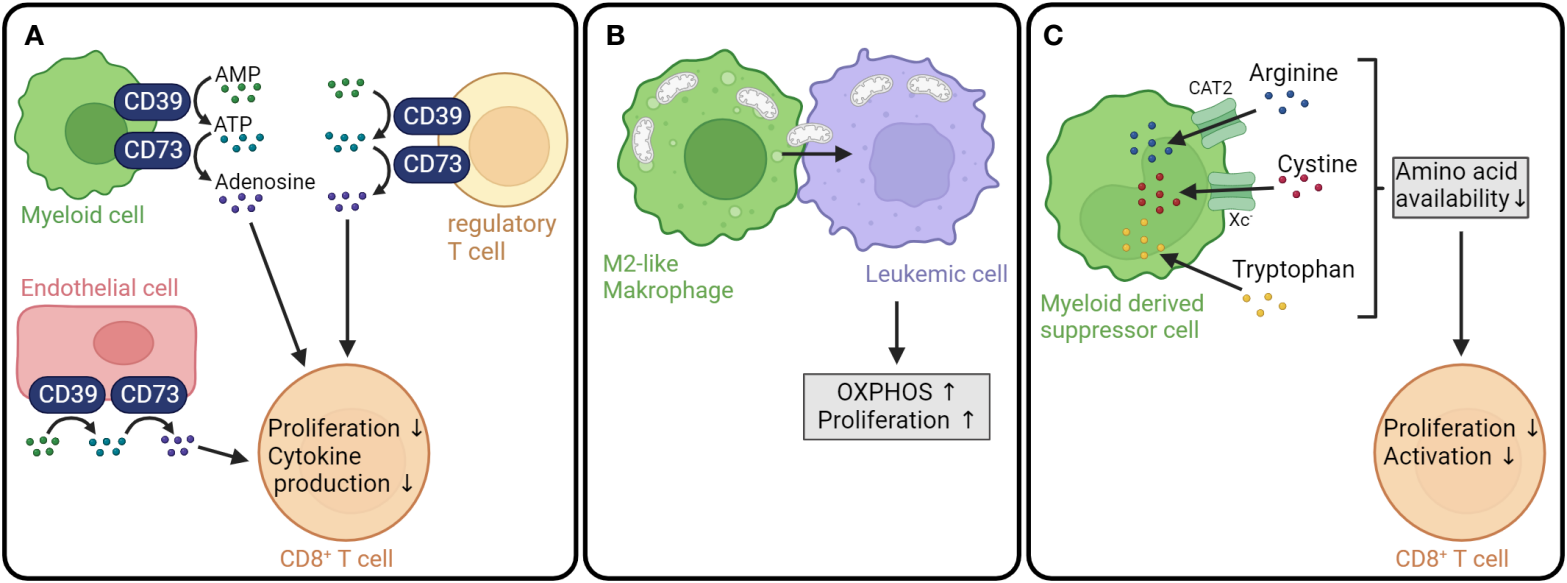
Myeloid cell metabolism influences GVL effect. **(A)** The membrane-bound ectonucleotidases CD39 and CD73 are expressed on a variety of cell types, including myeloid cells, T_regs_ and endothelial cells. CD39 converts ATP into AMP, which is subsequently dephosphorylated to adenosine by CD73. Adenosine inhibits T cell proliferation and cytokine production (167–169). **(B)** Tumor-supporting M2-like macrophages transfer mitochondria to leukemic cells resulting in increased OXPHOS and proliferation of leukemic cells (170). **(C)** Myeloid-derived suppressor cells import arginine, cystine and tryptophan leading to a decreased availability of amino acids crucial for CD8^+^ T cell proliferation and activation (171–174). AMP, adenosine monophosphate; ATP, adenosine triphosphate; OXPHOS, oxidative phosphorylation; CAT2, cationic amino acid transporter; Xc^-^, cystine/glutamate antiporter.

Not only leukemic cell metabolism, but also the metabolism of myeloid cells, such as tumor-associated macrophages (TAMs) and myeloid-derived suppressor cells (MDSCs) can shape the tumor microenvironment. One mechanism by which myeloid cells contribute to the inhibition of anti-tumor responses is the expression of the membrane-bound ectonucleotidases CD39 and CD73, which catalyze the production of the immunosuppressive metabolite adenosine (165, 166) (**Figure 4A**). CD39 converts ATP into AMP and is expressed by a variety of cell types including macrophages and dendritic cells (175, 176). CD73, in turn, dephosphorylates AMP to adenosine and is widely expressed on different cells and tissues (177, 178). *In vitro* differentiated human M2-like macrophages showed a higher expression of CD39 and CD73 compared to monocytes or macrophages differentiated into M1-like phenotype (179). Moreover, IL-27 secreted by tumor-infiltrating neutrophils has been found to drive CD39 expression in macrophages. Neutralization of IL-27 with an anti-IL-27 antibody resulted in a downregulation of CD39 and PD-L1 expression and a decreased IL-10 secretion by M2 polarized macrophages *in vitro* (180). Besides myeloid cells, several other populations can express CD39 and/or CD73, e.g., tumor cells (181–183), mesenchymal stromal cells (184, 185), endothelial cells (186), and T_regs_ (187). In a nontransplant setting, CD39 was found to be expressed on AML cells and T_regs_ from AML patients and to promote an immune-suppressive microenvironment (166). Interestingly, mutations in the key myeloid transcription factor C/EBPα were defined as a driver for elevated CD73 expression in a mouse AML model with an endogenous C/EBPα mutation (188). Taken together, myeloid cells together with tumor cells and bystander cells regulate the adenosine levels in the tumor microenvironment.

The balance between ATP and adenosine is important for immune homeostasis. ATP released from dying cells represented a danger signal and activated the immune system via binding to the purinergic receptor P_2_X_7_ on dendritic cells in mouse models of allo-HCT (165). In contrast, adenosine suppresses the antitumor activity through activation of G-protein coupled adenosine receptors (A_1_, A_2A_, A_2B,_ and A_3_-AR), with A_2A_ being the receptor predominantly expressed on T cells (189, 190). The binding of adenosine to the A_2A_-AR was demonstrated to inhibit mouse T cell proliferation, production of proinflammatory cytokines, and cytotoxicity (167, 191, 192) (**Figure 4A**). Systemic inhibition of CD73 in a preclinical B cell lymphoma/allo-HCT model resulted in improved T cell proliferation, cytokine production, and migration and boosted the GVL effect in an A_2A-_AR-dependent fashion (168, 169).

#### 5.3.2 Mitochondrial transfer

It has recently been shown that tumor-supporting anti-inflammatory macrophages can promote leukemic cell metabolism through mitochondrial transfer. A comparison of the proteome of AML patients with a high and a low percentage of M2 macrophages showed that leukemic blasts in the bone marrow of patients harboring a high amount of M2 macrophages were enriched in proteins involved in OXPHOS. When co-cultured with M2d macrophages, leukemic blasts displayed a higher oxygen consumption rate and mitochondrial ATP production. *In vitro* co-culture experiments with primary AML cells and mitochondria-labeled M2d macrophages revealed that AML cells take up mitochondria from the macrophages (170). Previously, the transfer of mitochondria from bone marrow stromal cells to AML cells had been described (193, 194). The elevated OXPHOS due to the increased number of mitochondria not only fueled the proliferation of leukemic cells (170), but also contributed to an enhanced chemotherapy resistance (193) (**Figure 4B**).

#### 5.3.3 Depletion of Amino acids

MDSCs might negatively influence the GVL effect by depleting amino acids required for T cell function through similar mechanisms as AML cells. When recruited to tumor sites, MDSCs upregulate cationic amino acid transporter 2 (CAT2) and arginase 1. The import of arginine through CAT2 and subsequent conversion into urea and L-ornithine by arginase 1 can lead to a depletion of arginine in the extracellular space resulting in impaired T cell proliferation and function (171). Moreover, MDSCs can uptake cystine and convert it to cysteine. However, since MDSCs do not express neutral amino acid transporter, they are not able to export cysteine resulting in a deprivation of cysteine, which is required for T cell activation (172). Additionally, MDSCs in breast cancer and chronic lymphocytic leukemia were shown to express high levels of IDO contributing to the depletion of tryptophan (173, 174). Taken together, MDSCs could contribute to an immunosuppressive tumor microenvironment by depleting arginase, tryptophan, and cysteine (**Figure 4C**). All mechanisms have so far been shown outside the context of AML, which is why their importance in regulating anti-leukemia immunity need to be investigated in the future.

### 5.4 Systemic metabolic events regulate anti-tumor immunity

#### 5.4.1 Oxidative damage/redox balance of T cells

Besides these examples of how AML metabolism and myeloid cell metabolism might directly impair the T cell function, several metabolic processes unrelated to AML itself also influence the GVL effect. The redox balance plays a crucial role in the function and regulation of alloreactive T cells. The increased metabolic activity upon T cell activation is accompanied by an increased generation of ROS (195). ROS arise from an electron leakage in mitochondrial complexes I and III, NADPH oxidases, xanthine oxidases, and several other enzymes (196, 197). Under normal conditions, T cells tightly control ROS concentrations with specific enzymes and endogenous antioxidants (198). Superoxide dismutase catalyzes the conversion of two superoxide anions into oxygen and H_2_O_2_, which is subsequently converted into water and oxygen by the enzyme catalase. Furthermore, glutathione peroxidase catalyzes the degradation of hydrogen peroxide and organic peroxides to alcohols (197). Besides, nonenzymatic small molecule antioxidants, in particular glutathione, play a role in the detoxification of ROS. Reactions with ROS oxidize glutathione, whereas the reduced form is regenerated by an NADPH-dependent reductase (199). While low to moderate ROS levels are essential for cell survival and proliferation, high levels of ROS harm the cell by causing DNA mutations, altering lipid metabolism, and inducing cell death (200).

**Figure 5:**
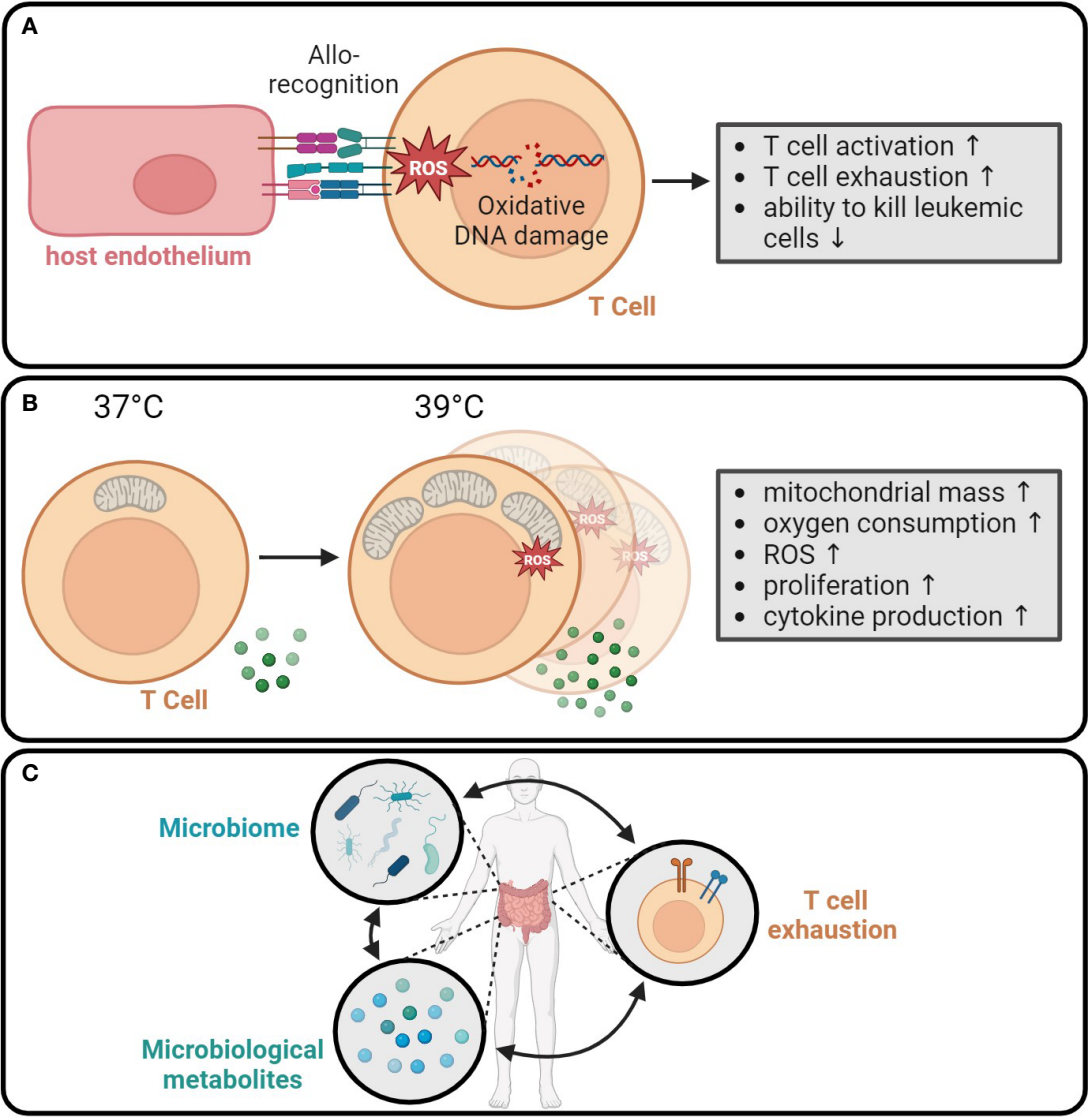
Systemic metabolic events regulate anti-tumor immunity. **(A)** Allorecognition leads to the production of ROS in T cells. The resulting oxidative DNA damage enhances short-term T cell activation, while increasing the T cell exhaustion and reducing the ability to kill tumor cells (204). **(B)** Fever causes an increase in mitochondrial mass, oxygen consumption, and ROS production in T cells. Moreover, the proliferation and cytokine production are enhanced at 39°C compared to 37°C (206). **(C)** Microbiome and secreted immunomodulatory metabolites interact with circulating T cells and probably contribute to T cell exhaustion (207).

Early studies have reported a disturbance in the redox balance in allo-HCT recipients. While the plasma levels of malondialdehyde and nitric oxide increased after allo-HCT, the activities of superoxide dismutase, glutathione peroxidase, and catalase decreased (201, 202). Investigation of the interaction between the host endothelium and alloreactive donor lymphocytes revealed that *in vitro* allorecognition induced genomic alterations in the epithelium through a ROS-mediated mechanism (203). A recent study confirmed that allorecognition itself contributed to oxidative DNA damage. Measurement of the oxidative DNA stress biomarker marker 8-hydroxydeoxyguanosine (8-OHdG) via ELISA showed that the concentration of 8-OHdG was elevated in serum, T cells, and NK cells of patients until day 60 after allo-HCT (n=50) but not in patients who underwent autologous stem cell transplantation (n=16). 8-OHdG^high^ T cells, on the one hand, were more proliferative and showed higher expression of the T cell activation markers CD25, CD69, and CD137 compared to 8-OHdG^low^ T cells. On the other hand, 8-OHdG^high^ T cells expressed higher levels of the T cell exhaustion marker PD-1 and KLRG-1, lower levels of IFN-γ, and were less efficient in killing AML cell lines and primary AML blasts, showing that oxidative damage impaired the GVL effect. Consistent with this hypothesis, high 8-OHdG levels in the T cells of patients after allo-HCT were associated with an increased relapse rate and a shorter overall survival (204). These findings raise the question of whether therapy with antioxidants could improve the function of allogeneic T cells. The treatment of CD8^+^ T cells *in vitro* with the antioxidant N-acetylcysteine resulted in a higher proportion of T cells with a stem cell memory-like phenotype. In a chimeric antigen receptor (CAR) T cell mouse model, the N-acetylcysteine-pretreated CAR T cells showed superior antitumor efficacy compared to CAR T cells cultured in the absence of N-acetylcysteine (205). In summary, these findings underline the importance of redox balance for allogeneic T cells and provide a rationale for targeting oxidative stress in T cells to improve their anti-tumor function (**Figure 5A**).

#### 5.4.2 Fever

Fever is a systemic reaction associated with multiple inflammatory conditions. Increased body temperature regulates metabolism, for instance, by altering the activity of temperature-dependent enzymes. Recent research has focused on how fever affects T cell metabolism and immune responses. Mouse CD8^+^ T cells, which were transiently exposed to 39°C (instead of 37°C) culture conditions, showed increased mitochondrial translation, mitochondrial mass, and oxygen consumption, resulting in improved cytokine production and long-term function (**Figure 5B**). When such T cells were adoptively transferred in a preclinical AML/allo-HCT model, they showed an enhanced capacity to eliminate leukemic cells, resulting in prolonged survival of the mice (206). More recently, CD4^+^ T cell lineages were found to have a differential sensitivity to fever. Exposure of mouse CD4^+^ T cells to 39°C *in vitro* boosted their proliferation and elevated the production of pro-inflammatory cytokines such as IFN-γ and IL-17. Simultaneously, induced T_reg_ in these conditions had an impaired suppressive capacity in *in vitro* co-cultures with cytotoxic CD8^+^ T cells. These data provide evidence that heat benefits pro-inflammatory CD4^+^ T cell responses. However, this study also showed that Th1 cells were particularly sensitive to increased temperatures, as they accumulated high levels of mitochondrial ROS, resulting in DNA damage and cell death (208). These preclinical studies provide insight into how systemic factors, such as the organismal temperature, might affect the GVL immunity.

#### 5.4.3 Microbiome

Another example of how systemic metabolism might regulate anti-leukemia immunity was provided by a recent study, which integrated intestinal microbiome and metabolome analysis with immune phenotypes to study the outcome of allo-HCT recipients treated with the antibiotic azithromycin (209). Azithromycin is a second-generation macrolide with efficacy in patients with bronchiolitis obliterans, a lung transplantation-associated condition of the lung with similar features to chronic lung GVHD (210). A clinical trial that evaluated the efficacy of azithromycin to prevent chronic lung GVHD surprisingly found that azithromycin-treated patients had a higher risk of malignancy relapse (207). In a complex analysis of the fecal microbiome, fecal and plasma metabolome, and circulating T cell immune phenotypes, the authors showed that the fecal microbiome of post-allo-HCT patients (n=55) can be divided into four different enterotypes. These enterotypes were associated with specific metabolic changes both in fecal and peripheral blood samples. Notably, taxa related to *Bacteroides fragilis* correlated with circulating exhausted T cells expressing TIGIT, PD-1, and TOX (207) (**Figure 5C**). Microbiome composition has been shown to directly correlate with the incidence of GVHD (211, 212), at least partially through the production of immunomodulatory metabolites, such as butyrate (213). The relationship between microbiota composition and anti-tumor immunity is still not well understood and warrants further studies.

## 6 Discussion

In recent years, considerable progress has been made in deciphering the mechanisms by which metabolic processes instruct T cell functionality. In the same time, our understanding of how AML cells shape the metabolic BM environment, and consequently allogeneic T cell immunity, is still developing. It has been uncovered that AML-derived metabolic products, such as lactate and R-2-HG, have an immunosuppressive effect on T cells and thus might promote immune escape after allo-HCT. Furthermore, there is increasing evidence that the metabolism of AML cells contributes to the upregulation of immune checkpoint ligands, which inhibit T cell activation. Additionally, systemic metabolic events, such as fever or oxidative damage caused by alloreactivity itself, were shown to influence the T cell activity. The identified mechanisms of how the GVL activity is modulated by metabolism open new possibilities for therapeutic targeting. Improving the redox balance of alloreactive T cells using antioxidants (204) and reversing the lactic acid-induced inhibition of glycolysis in T cells (130) are two of the most promising options. New treatment options are urgently needed since the rate of AML patients relapsing after allo-HCT is still high (4). Nevertheless, most insights about the potential metabolic regulation of the GVL immune response stem from preclinical or *in vitro* studies and are partly extrapolated from other diseases. Defining which of these metabolic alterations recurrently happen in allo-HCT recipients and how they contribute to immune evasion is one of the essential tasks in the field.

We believe that future studies could focus on several biological aspects. First, the importance of the local metabolic niche (in the BM) versus systemic metabolism (in the peripheral blood) is not understood. The BM niche is a hypoxic environment (214), and it is likely that oxygen tension is even lower in the presence of an overt leukemic cell population. Additionally, little is known about how the BM concentrations of polar metabolites and lipids differ from their plasma levels. Recent studies in the metabolism field have uncovered that the metabolite concentrations in frequently used cell culture media differ sometimes substantially from their plasma concentrations and that utilizing media with “physiological” metabolite levels uncovers novel aspects of cancer and immune cell metabolism (215–218). It is likely that local metabolic conditions in the BM affect immune cell function, and further studies are required to define the BM niche metabolic composition in health and disease. Furthermore, it is possible that microbiota-derived metabolic products shape the phenotype and function of alloreactive T cells. In the context of GVHD, it is now established that microbiota-derived products, such as butyrate, affect intestinal inflammation (219, 220). Recent evidence suggests that distinct bacterial enterotypes are associated with alterations in the fecal and plasma metabolome, and consequently can influence T cell function (209). Further studies are warranted to dissect the contribution of individual (microbiota-derived) metabolites on the systemic level to anti-leukemia immunity.

Several studies discussed above focus on how leukemia cells might suppress the immune response by altering the concentration of metabolites in the microenvironment and thus impairing T cell activity by nutrient depletion or toxic metabolite accumulation. However, how metabolic processes affect the recognition of AML cells by the immune system has not been reported. In other tumors, a correlation between MHC expression and metabolism was found (221, 222). Inhibition of dihydroorotate dehydrogenase (DHODH), an enzyme essential for *de novo* pyrimidine synthesis, was shown to upregulate the expression of MHC class I in pancreatic tumor cell lines and the melanoma cell line B16F10 (221). DHODH inhibitors have demonstrated anticancer activity in different preclinical tumor models (223–225), including AML (226), and are currently tested in a phase I trial for patients with acute myeloid leukemia and myelodysplastic syndrome (NCT04609826). Since the downregulation of MHC represents a mechanism of immune escape in AML (27), future work could investigate how MHC expression is regulated by AML cell metabolism. Moreover, emerging evidence indicates that metabolic processes can drive the expression of inhibitory checkpoint ligands on AML cells, but further studies are required to understand whether this plays a role for post-allo-HCT relapses.

Broadly speaking, while most studies focus generally on how leukemia metabolism might affect T cell function (78, 79, 135, 166), very little published research has focused on mechanisms specific to the post-allo-HCT relapse setting in humans (130). It is likely that metabolic adaptations occur as the disease progresses and that relapsing AML after allo-HCT imposes unique metabolic constraints on allogeneic T cells. The specific nature of this metabolic reprogramming remains still to be understood.

Overall, more research is needed to examine the complex interaction between AML cells, T cells, and other cells present in the tumor microenvironment. Although they were not the primary focus of this review, other immune cells, such as macrophages and NK cells, are also influenced by AML metabolism. For instance, the secretion of arginase II by leukemic cells plays a role in the polarization of surrounding monocytes into tumor-supporting “M2-like” macrophages (78). These tumor-supporting macrophages can likewise contribute to the immunosuppressive tumor microenvironment, for example by expressing CD39 and CD73, which convert ATP into immunosuppressive adenosine (175, 176). In addition, the metabolism of AML cells additionally affects non-immune cells. BM adipocytes, for example, were demonstrated to release fatty acids after induction of phosphorylation of hormone-sensitive lipase by AML blasts (83) and BM stroma cells were found to transfer mitochondria to AML cells (193, 194). The immune and non-immune cells modulated by AML metabolism can, in turn, interact with the T cells and thus affect their activity. Understanding the metabolic interplay of all different cell types holds the potential to inform novel therapeutic developments.

## Author contributions

A-CB: Conceptualization, Investigation, Writing – original draft, Writing – review & editing. PA: Conceptualization, Writing – original draft, Writing – review & editing.
